# Sequential Soil Transport and Its Influence on the Spatial Organisation of Collective Digging in Leaf-Cutting Ants

**DOI:** 10.1371/journal.pone.0057040

**Published:** 2013-02-15

**Authors:** Steffen Pielström, Flavio Roces

**Affiliations:** Behavioral Physiology and Sociobiology, Biocenter, University of Würzburg, Würzburg, Germany; Arizona State University, United States of America

## Abstract

The Chaco leaf-cutting ant *Atta vollenweideri* (Forel) inhabits large and deep subterranean nests composed of a large number of fungus and refuse chambers. The ants dispose of the excavated soil by forming small pellets that are carried to the surface. For ants in general, the organisation of underground soil transport during nest building remains completely unknown. In the laboratory, we investigated how soil pellets are formed and transported, and whether their occurrence influences the spatial organisation of collective digging. Similar to leaf transport, we discovered size matching between soil pellet mass and carrier mass. Workers observed while digging excavated pellets at a rate of 26 per hour. Each excavator deposited its pellets in an individual cluster, independently of the preferred deposition sites of other excavators. Soil pellets were transported sequentially over 2 m, and the transport involved up to 12 workers belonging to three functionally distinct groups: excavators, several short-distance carriers that dropped the collected pellets after a few centimetres, and long-distance, last carriers that reached the final deposition site. When initiating a new excavation, the proportion of long-distance carriers increased from 18% to 45% within the first five hours, and remained unchanged over more than 20 hours. Accumulated, freshly-excavated pellets significantly influenced the workers' decision where to start digging in a choice experiment. Thus, pellets temporarily accumulated as a result of their sequential transport provide cues that spatially organise collective nest excavation.

## Introduction

When excavating subterranean nests, animals have to dispose of the removed soil by transporting it from the underground to the surface [1]. With increasing nest size and depth, builders invest a large proportion of their time and energy budget into soil transport, especially when underground nests are not built by solitary individuals, but constructed collectively by animal societies. In termites and ants for instance, the constructions can reach gigantic dimensions as compared to the body size of the builders, and the amounts of soil to be relocated as well as the transport distances are substantial [2], [3]. However, the organisation of underground soil transport in animal societies remains largely unexplored, with the exception of a detailed study on soil transport in the naked mole rat *Heterocephalus glaber* Rüppell, in which workers were observed to utilize sequential task partitioning to move soil to the surface [4]. The term task partitioning denotes a process in which a single task, e.g. the transport of one particular food item or soil particle, is split up between different worker groups [5]–[8].

Despite the fact that ant colonies move considerable amounts of soil while excavating underground nests, published works on ants refer only to the organisation of soil disposal and the emergence of mounds around the nest entrances (see references in [9]), with some early observations on task partitioning during the deposition of soil at the surface in *Cataglyphis longipedem* (Eichwald, formerly known as *Myrmecocystus viaticus setipes* Forel) and *Messor barbarus* (L.) [7], [10]. One ant species that has been studied intensely in terms of construction and functionality of external nest architecture is the leaf-cutting ant *Atta vollenweideri* (Forel) from the Gran Chaco region in South America. Workers of this species usually build turrets on top of the nest openings in the centre of their nest mound. These turrets, which enhance wind-induced nest ventilation [11], [12], are mainly built with materials collected from the soil deposits around the nest openings, i.e. materials previously transported to the surface from the underground or collected from adjacent areas [9], [13]. In colonies of this species, the effort made to relocate soil is expected to be particularly high. The nests of *A. vollenweideri* are among the largest structures built by social insects. At a depth of around 1–2 m, workers excavate thousands of closely-packed fungus chambers in order to contain the symbiotic fungus as well as the queen and brood. At deeper soil layers, larger chambers are excavated and used as deposits for the colony refuse. An intricate arrangement of tunnels connects the underground chambers to the surface, where the soil excavated from underground is heaped in a mound that can be 1 m high and 8 m wide in a mature nest [14], [15]. The sheer amount of soil colonies of this species move from deeper layers to the surface makes them a major factor shaping the composition of the vegetation in the Gran Chaco region [16]–[19].

In a nest of these dimensions, with transport performed over large distances and against gravity, workers are expected to incur large time and energy costs in carrying soil. Similar to desert ants that are known to lift and carry sand with the aid of their psammophores or even maxillary palps [20], [21], [22], *A. vollenweideri* workers actively form aggregations of fine material [13], so called ‘pellets’ [23], but carry them in between the mandibles without the aid of any specialised structures. The transport rates of soil pellets, which can vary in size considerably, may be higher if size matching occurs between worker size and load size, as known for foraging ants and even *A. vollenweideri* workers excavating soil to built ventilation turrets [9], [24], [25]. In addition, it is unclear if an ant excavating soil will be able to travel across the nest to deposit the excavated load outside, and then once again find the excavation site in the complex nest structure. The distances between an excavation site and the closest nest opening can be expected to regularly exceed 1–2 m, and the way may involve several tunnel forks.

In the context of long-distance leaf carriage, *A. vollenweideri* and other *Atta* species have been reported to rely on sequential transport. Leaf fragments are often not carried into the nest by the cutting individual, but dropped on the foraging trail where they are picked up by other workers [26]–[29]. Sequential transport of soil loads, as known in the context of leaf carriage, may provide advantages regarding the spatial orientation of the involved workers, as compared to an individual mode of transport. Interestingly, accumulation of soil pellets inside leaf-cutting ant nests has regularly been observed [14], [15], [30]–[32], suggesting that excavators do not necessarily carry the excavated soil to the outside, and that sequential soil transport may occur under natural conditions. If temporary deposits of freshly excavated soil occur, accumulated soil pellets may provide local information to other workers about current digging activity in the vicinity, thus acting as stigmergic cues that may lead arriving workers to search for the digging site. Stigmergy is a regulatory process aimed at explaining how collective building behaviour can be organised without central control [33], [34]: by changing the environment through building behaviour, the structure created by one individual passively provides a cue that influences the behaviour of other individuals, i.e. coordination is achieved through the structure workers are actually building. When deciding where to excavate, workers of *A. vollenweideri* might use pellets deposited by other excavators as a cue. Thereby, sequential soil transport may be beneficial not only for the orientation of the carriers, but also for the spatial organisation of further excavation activity.

The aim of this study was to characterise the transport of soil pellets in the leaf-cutting ant *A. vollenweideri*, and to investigate whether soil pellets are temporarily deposited inside the nest and influence the excavation behaviour of nearby workers. In a first experiment, pellet mass was compared to carrier body mass to investigate a possible size matching. Then, the occurrence of sequential transport was quantified under controlled laboratory conditions by observing soil pellets from the excavation site until they were deposited outside the nest. The behaviour of individual excavators was observed in a separate experiment, measuring how fast they produced pellets and observing where they deposited them. The response of other excavators to deposited pellets was evaluated in three different choice experiments, aimed at investigating whether single, fresh and old soil pellets influence the spatial decision of workers as where to initiate digging.

## Methods

### Animals and material

*A. vollenweideri* is a widespread species in the Gran Chaco Region. It is not protected under the Convention on International Trade in Endangered Species (CITES) and no specific permits are required for the described studies. The study site in the Reserva El Bagual, San Francisco de Laishi, Formosa, Argentina (26°17′08″ s; 58°49′43″ w) is privately owned by the Estancia EL BAGUAL-ALPARAMIS S.A. The research was conducted with the permission of Pablo Götz (owner) and Alejandro Di Giacomo (supervisor of the ‘Reserva’). Three different large laboratory colonies were used during the experiments, all of them excavated at an age of approximately 8 months in 2004. Colonies were kept under controlled conditions at 25°C and a 12∶12 h LD cycle. For laboratory experiments, standardized industrial clay (CLAYTEC ‘Lehm gemahlen 10.001’) with a maximum particle size of 0.5 mm was mixed with water. In series using a mix of clay and sand, standard playground sand (Red Sun garden products, article no. 1110116) was used. Statistical analyses were done with R 2.14.1 (http://www.R-project.org).

### Size matching during soil transport

To characterise soil transport and size-matching in *A. vollenweideri*, workers carrying soil pellets were collected in the field and in the laboratory, and both body mass and load mass were measured at the nearest 0.1 mg. In the field, samples were collected at nest openings, where the carriers were expected to have already covered a transport distance of up to several meters. In the laboratory, two different samples of loaded workers were taken. In the first sample, loaded workers were collected after having walked through a 2 m long tube. Since workers were not individually marked, it was unknown whether the observed carriers excavated their own pellets, or collected pellets previously excavated by other workers. In the second sample, pellets and the corresponding excavators were collected directly at the excavation site. Thus, the different samples allowed size-matching between loads and body masses to be comparatively analysed for both excavators and carriers.

Size-matching in the field was investigated during a field trip on November 17^th^, 2008. At the Reserva El Bagual, San Francisco de Laishi, Formosa, Argentina, 7 mature field nests were selected from three different areas at a maximum distance of 1.5 km from each other. At each nest, a random sample of soil carriers was collected by selecting one opening, closely observing it and picking each individual coming out with a soil pellet by means of a forceps. Only 10 workers were picked from each nest to be able to move to the next nest in time, and thus to sample all the nests the same day. Workers still holding their pellets were immediately transferred into Eppendorf vials. After collection, carrier body mass, wet pellet mass and dry pellet mass were weighed, and soil water content was calculated. Eleven samples were damaged during transport and therefore not considered for further analysis.

For the collection of loaded workers in the laboratory under conditions comparable to those in the field, two laboratory colonies were connected to open plastic boxes (9×9×6 cm), called ‘collecting boxes’, that allowed the collection of workers with forceps. From such a plastic box the ants were able to enter a 2 m long flexible transparent tubing with an inner diameter of 10 mm. At the distal end, the tube was connected to a second box of the same dimensions entirely filled with a soil mixture composed of 60% clay, 20% sand and 20% water. The colonies were connected at the evening and the next day, 40 carriers with their loads were collected from each colony after having walked over 2 m, transferred into Eppendorf vials, and weighed as described.

A comparable procedure was used to collect pellets and their excavators. The clay was placed in the collecting box to a depth of about 1 cm, and no tube and second box were necessary. Workers were selected while digging at the surface and caught with forceps after having finished a pellet. Again, 40 workers with their corresponding pellets were collected from each colony and transferred into Eppendorf vials for weighing. To evaluate the relationship between pellet mass and carrier body mass, both measures were used as variables in a regression analysis.

### Transport chains – ‘Pelletograms’

The second experiment was performed to characterise the entire transport process of a single pellet from excavation to deposition outside the nest. In analogy to an ethogram, which characterises all behaviours and movements observed in an animal within an observation period, we created so-called ‘pelletograms’ by observing single soil pellets and recording their transports, the number of carriers involved in the entire transport process, their waiting times, overall transport speed, as well as walking velocities and transport distances of the participating carriers.

To observe the entire transport of excavated pellets from excavation to their deposition outside the nest, a laboratory colony of *A. vollenweideri* was attached via a T-shaped connector to a box for pellet deposition at one side, and a 200 cm long transparent tube with an inner diameter of 10 mm on the other. At the distal end, the tube was connected to a second tube completely filled with moist clay (20% water content), so that the ants, when reaching the end of the tube, were able to excavate in clay. Along the way from the clay to the depositing box, a centimetre scale was drawn on the outside of the tube to allow for fast assessment of the position of dropped pellets.

Timing began with the beginning of excavation activity. Subcolonies were allowed to continuously excavate over two days, to evaluate whether the measured parameters change over time when the excavation site became known to the ants. For each observation, a digging worker was selected and the progress of its excavated pellet was followed. Every movement of that pellet was noted in terms of pick-up time and as well as dropping time and position, until it finally reached the depositing box. That way, for each pellet we monitored, how much time it had spent to cover a distance of 200 cm, how many workers had been involved in the transport process, how long it had lain on the way in between two transport events, and what distance had been covered at what speed in each transport event.

After starting the experiment, single pellets were observed over a period of 6 to 7 h. The next day, new clay was pushed into the tube from the distal end before continuing the observations, to keep the distance from the digging face to the depositing box constant, i.e. to compensate the tunnelling progress the ants had made over night. The whole procedure of offering an excavation site previously unknown to the ants, i.e. of connecting a fresh tube leading to a new excavation site to the colony, and observing pellets during their transport process for two days, was repeated six times, each experiment yielding observational data for about 20 pellets.

To test for temporal changes in the observed parameters, the time since the experiment had been started was taken as the predicting variable in regression analysis. Transport duration, number of carriers involved and waiting time were each *ln* transformed to achieve normal distribution of residuals, and each day was tested separately. Three different functional worker groups were distinguished: excavators, defined as the workers carrying their own excavated pellet; short-distance carriers, defined as the workers that pick up a pellet and carry it for a distance, but not out into the depositing box; and long-distance carriers that cover the last part of the distance and deposit the pellets outside. These functional groups were analogous to those described during sequential transport of leaves in foraging ants: cutters, which cut the leaves and often carry the fragments only for short distances, short-distance carriers that drop their fragments on the way, and long-distance carriers that cover the remaining distance to the nest [28].

The three groups were, again day wise, compared in terms of their walking speed and the distance they covered. While the former comparison was done using an ANCOVA, the latter failed to show normally distributed residuals even after transformation due to the strong bifurcation of the data.

Therefore, we utilized a resampling technique to compare the groups [35]. Confidence intervals for the means and regression coefficients were bootstrapped by recalculating both values for 10^4^ random subsamples of *N* = 20 each. From those distributions, quantiles were calculated to determine confidence intervals. The effect of time was considered significant when the estimated 95% confidence intervals of the regression coefficients did not include 0. To compare the means of all groups with each other, three inter-group comparisons were necessary. Therefore, the significance level *α* was corrected from 1/20 to 1/60. The 98.3% confidence intervals were estimated for all means. Mean values with overlapping 98.3% confidence intervals were considered not significantly different from each other.

Finally, the percentage of long-distance carriers within all carriers, i.e. long-distance plus short-distance carriers, was calculated for each hour. A generalized linear model (GLM) with quasibinomial error distribution was used to analyse changes of this percentage as a function of time.

### Quantifying pellet excavation – Ethograms

The observation of individual workers excavating soil pellets was conducted with subcolonies, i.e. groups of workers without a queen, separated from the rest of the colony, but maintaining their own fungus garden. The subcolonies were allowed to excavate in a thin layer of soil between two horizontal glass plates. The setup made it possible to directly observe individually marked workers over an extended period of time, with the aim of assessing how long a single worker is engaged in digging behaviour, how many soil pellets it can form in that time, and where it deposits the pellets after producing them.

**Figure 1:**
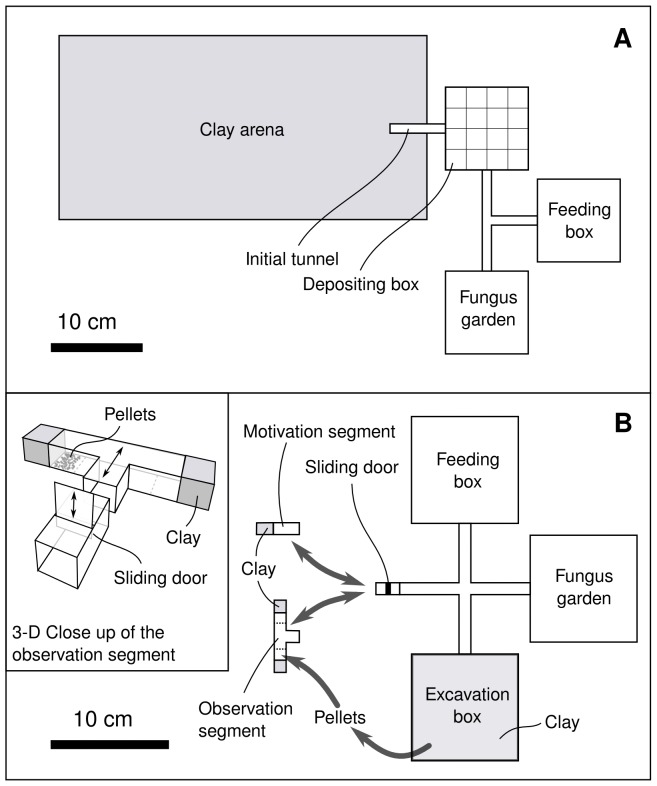
Experimental setups for ethograms and choice experiments. **(A)** Setup for observations on individual workers while excavating in soil and depositing pellets. Observations of individually marked workers were done both inside the clay arena, where soil pellets were excavated, and in the depositing box, where the most pellets were deposited. **(B)** Setup for choice experiments to test the influence of deposited pellets on the workers' decisions where to excavate. The motivation segment was connected to the subcolony to stimulate ants to excavate at that location, while soil pellets from the excavation box were placed at one side in the observation segment for the subsequent choice situation. After replacing the motivation segment with the observation segment (inset), it was observed at which side the ants started to dig first.

The subcolonies were assembled in small transparent plastic boxes (9×9×6 cm) connected to the laboratory colony during the weeks before the experiments. In this time, workers filled the boxes with cultivated fungus. These boxes, henceforth referred to as ‘fungus gardens’, were separated from the main colonies and opened to remove all workers but the fungus gardeners. Afterwards, each fungus garden was connected with a T-shaped tube (10 mm inner diameter) to two additional boxes of the same size, one daily replenished with fresh leaves, water and 1∶1 honey water solution, from now referred to as the ‘feeding box’, the other one empty. The empty box was the ‘depositing box’, intended as a location for the ants to deposit soil pellets. The depositing box was later connected to the ‘excavation arena’, a 4 mm layer of soil composed of 60% clay, 20% sand and 20% water, placed between two horizontal glass plates of 400×200×3 mm each. The connecting tube from the depositing box directly lead into a pre-formed tunnel 1 cm wide and 4 cm long, leading from the centre of one short side of the excavation arena directly to the centre of the rectangle in an angle of 90°. This was the place where workers from the subcolony started excavating (Fig. 1A). Until the experiment started, the connection between depositing box and excavation arena was kept closed.

For each subcolony, 200 intermediate size workers were chosen from the main colony and added to the separated fungus garden. Seventy-five of these workers were individually marked using a colour code based on three dorsal marking spots on the ant's body: one on the posterior part of the head, the second on the two anterior segments of the mesosoma, the third on the two anterior gaster tergites. The colours used were from Edding-750 markers, applied to the marking spots by means of the tip of a thin steel wire. After assembling, the subcolonies were given time to calm and familiarize with the new environment for two days. On the third day, experiments were started.

Each experimental day started with the preparation of a new excavation arena and its connection to the subcolony. Dead individuals were replaced to keep group size and proportion of marked individuals constant. When the ants had started to dig in the excavation arena, one marked individual entering the arena and joining the excavation was chosen for close observation. Behaviours were recorded as a real-time ethogram in the software BioLogic (v. 1.0.0). Four different types of events were defined in the program: ‘start digging’ to denote that the observed individual had started to excavate, ‘stop digging’ for the moment the ant ceased excavation, and ‘pick’ and ‘drop’ to record pellet transportation activity the same way. This way it was possible to record when and how long an individual was engaged in either pellet digging or pellet carrying. Additionally, the location where pellets were dropped was recorded, distinguishing ‘arena’ for the excavation arena, ‘tube’ for the connecting tube and ‘box’ for the depositing box. The floor area of the depositing box was itself divided into 16 equal squares. If a pellet was deposited there, it was noted in which of the squares the pellet had been dropped. This allowed for the calculation of the Index of Dispersion for each individual, to evaluate whether a worker drops its excavated pellets in an equal-, random- or aggregated distribution.

On each day, observations were conducted for up to 7 h, and each worker was observed for as long as possible. Only when leaving the arena and depositing box for the fungus garden or feeding box and staying absent for more than 10 min, observations for this worker were finished, and another one was chosen. The observation of an individual was not considered for analysis if the worker did not reach the termination criterion of 10 min absence until the end of the day. However, this was never the case. Twelve individuals from two different subcolonies were observed. Indices of Dispersion were calculated for 8 workers, because workers were included in the analysis only if the exact locations of all their dropping events could be recorded.

### Dropped pellets as cues for digging decisions – Choice experiments

To investigate whether deposited pellets act as cues that attract workers and spatially guide their decision where to dig, two alternative locations to excavate were offered simultaneously in a choice experiment, one with pellets deposited in front of the soil face, the other without. To avoid disturbance or overcrowding due to the separation from the colony, subcolonies were allowed to excavate at a particular spot that was later replaced by a T-shaped choice setup offering two alternative locations to excavate (Fig. 1B). In this set-up, small groups of 5–6 animals were separated from the tube leading to the subcolony by using a sliding door, so as to avoid overcrowding. Only the first reaction of one of the 5–6 ants, i.e. the first occurrence of digging behaviour at one of the two alternative sites, was counted as a response. The measured responses can therefore be considered as individual choices rather than group responses, even though the choosing individual was not completely isolated from other workers.

Three different conditions were tested. In the first, the whole ground on one side was covered with freshly-excavated pellets. In the second, the pellets were stored for one hour before the experiment, to allow potential chemical cues to evaporate, and offered on the whole ground as in the previous condition. In the third condition, a single fresh pellet was offered on the ground.

The subcolonies used for the experiments were created by separating a small fungus box including workers from the main colony and connecting it to a cross-shaped tube. Through that tube, the ants could either enter a feeding box via one direction, or, in the opposite, a similar sized box filled about 1 cm high with clay (18% water content), hereafter referred to as the ‘excavation box’. Workers excavating in that box provided the pellets used as a stimulus in the choice experiment. Pellets were collected from the excavation box using forceps, that were cleaned with 100% ethanol after each use, and stored into the ‘evaporation tube’, another tube of 10 mm inner diameter that was closed at one end using a plug with a small hole (about 3 mm diameter) in the centre. The other end was connected to a pump pushing air through the evaporation tube. In between the pump and the evaporation tube, a water bubbler was connected so as to saturate the circulating air. Hereby it was possible to allow chemicals on the pellets to evaporate and to prevent desiccation. Pellets for the ‘one hour’ condition were kept here for one hour, those used in the other conditions put into the evaporation tube as well to rule out any effects that may come due to the treatment itself, but were removed after only one minute. Moving straight ahead from the fungus garden, the tube was connected via a small sliding door to a tunnel section with a square-shaped cross section that had been created by removing the bottom from 4×1×1 cm photometric plastic vial. The end of this tunnel was plugged with another piece of clay. We will call this part of the setup the ‘motivation segment’ throughout the text. Here, as well as inside the excavation box, ant workers soon began to dig.

When digging activity was observed inside the motivation segment, the motivation segment was replaced with the ‘observation segment’, a T-shaped tunnel segment built from the same material, but branching to end in two equal clay walls located in opposite directions at a distance of 4 cm from each other. At one side, the square centimetre on the bottom immediately in front of the clay was covered with pellets from the excavation box. The clay surface was on both sides marked with an X-shaped scratch. Assuming that surface irregularities can motivate ants to start digging at them, as reported for termites [36], scratching the surfaces was aimed at shortening the response time of the workers to gain more replicates within the same time. A sliding door prevented additional workers from entering the segment. The fact that the observation segment replaced another segment in which workers had dug already ensured a number of workers with a high probability of resuming excavation behaviour to return to that location.

After connecting the observation segment to the setup, the sliding door was closed as soon as 5–6 workers had entered. Hereby, on the one hand, inconclusive observations caused by ants digging simultaneously at both alternative sides under crowed conditions were avoided; on the other hand the workers were not torn out of their social environment. Preliminary observations had shown us that individual workers completely isolated from nestmates wait much longer until they start excavating, if they excavate at all. The isolated worker groups in our experiment were observed for 10 minutes. As soon as the first of the observed workers started to excavate at one side, this was counted as a decision for that side. Workers were then removed from the observation segment and kept separately until the experiment was finished. The observation segment was again replaced by the motivation segment for a period of at least 15 minutes. The observation segment was cleaned with 100% ethanol and the side with the pellets switched after each observation.

The percentage of choices for the side with the pellets was tested for deviation from a theoretical 1∶1 distribution with a binomial test.

## Results

### Size matching during soil transport

**Figure 2:**
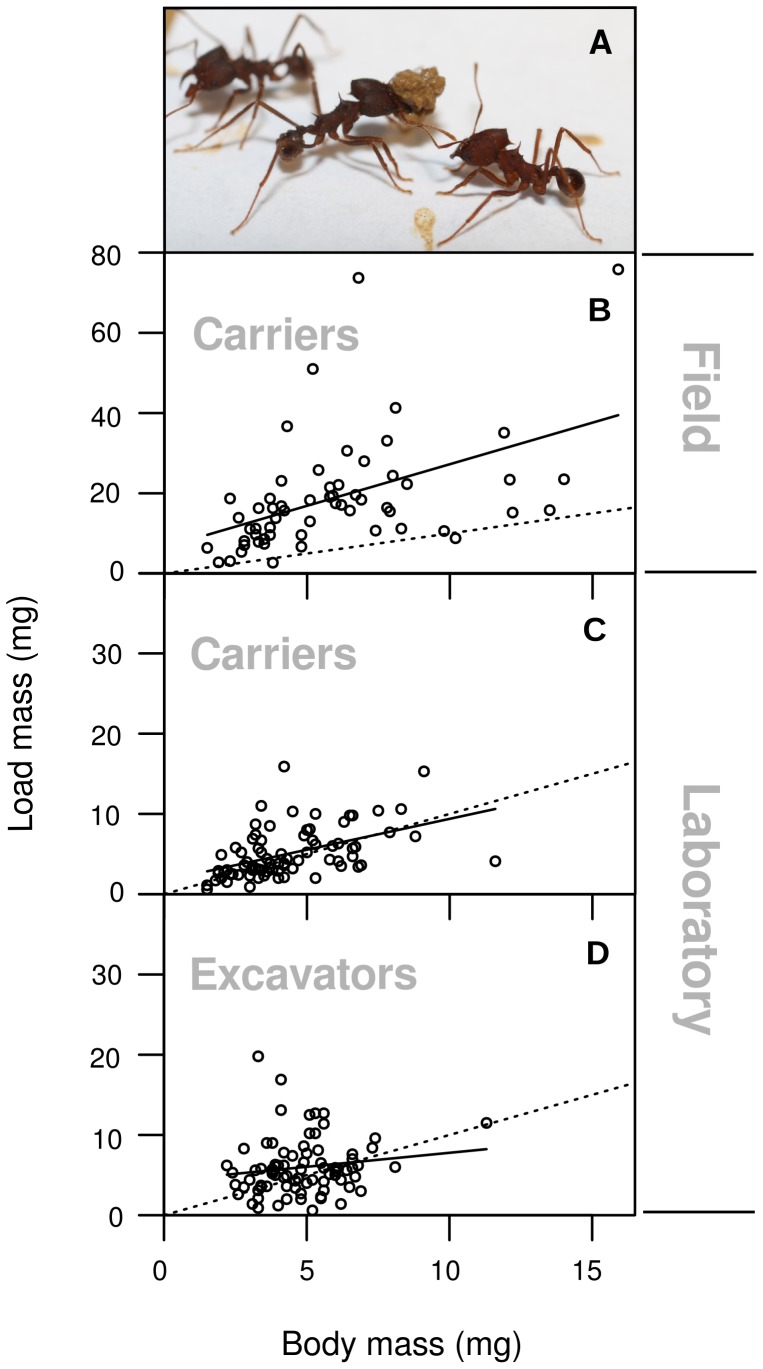
Relationship between pellet mass (m_P_) and worker body mass (m_W_). In each graph, the solid line represents the regression line and the dotted line a hypothetical 1∶1 ratio of load mass to carrier mass. Data points above that line correspond to carriers less heavy than their loads. **(A)** *A. vollenweideri* worker carrying a soil pellet in the laboratory (Photo: Andrew I. Bruce). **(B)** Pellets and carriers collected at the openings of field nests ( m_P_ = 2.1 m_W_+6.5, *r* ^2^ = 0.22, *N* = 60, *P*<0.001). **(C)** Pellets and carriers collected from laboratory colonies at 2 m distance from the excavation site ( m_P_ = 0.8 m_W_+1.7, *r* ^2^ = 0.24, *N* = 80, *P*<0.001). (d) Pellets and excavators collected directly at the excavation site ( m_P_ = 0.3· m_W_+4.3, *r* ^2^ = 0.02, *N* = 80, *P* = 0.1859).

Pellets collected in the field (Fig. 2B) ranged from 2.7 mg to 75.8 mg with an average mass of 15.8±12.1 mg, containing 14.0±5.0% water (median±interquartile range, *N* = 67). Pellets were predominantly carried by workers of intermediate size, the smallest worker weighing 1.5 mg, the largest 15.9 mg, the median being 5.2±4.0 mg (*N* = 67). Pellet size significantly depended on the size of the worker carrying it (*F*_1,57 = _16.21, *P*<0.001) with the carriers, especially the smaller ones, usually carrying more than their own body mass: the median loading ratio, i.e. the proportion of load mass to carrier mass was 2.9±2.1.

Carried pellets (Fig. 2C) collected in the laboratory at 2 m distance to the digging site had an average mass of 4.0±3.7 mg (median±interquartile range, *N* = 80), containing 18.8±0.1% (*N* = 80) water. The body mass of carrying workers was 3.9±2.2 mg (*N* = 80). Again, pellet size significantly depended on carrier size (*F*_1,78 = _24.09, *P*<0.001). Here, loading ratio was lower than in the field, with median of 1.0±0.7 (*N* = 80), indicating that workers carried loads roughly equivalent to their own body mass.

Freshly excavated pellets (Fig. 2D) weighed 5.5±3.8 mg (median±interquartile range, *N* = 80) with 20.9±0.1% (*N* = 80) water content. The body mass of the excavators was 3.8±1.8 mg (*N* = 80). Regression analysis showed no significant dependence of pellet size on excavator size (*F*_1,78_ = 1.78, *P* = 0.186).

### Transport chains – ‘Pelletograms’

**Figure 3:**
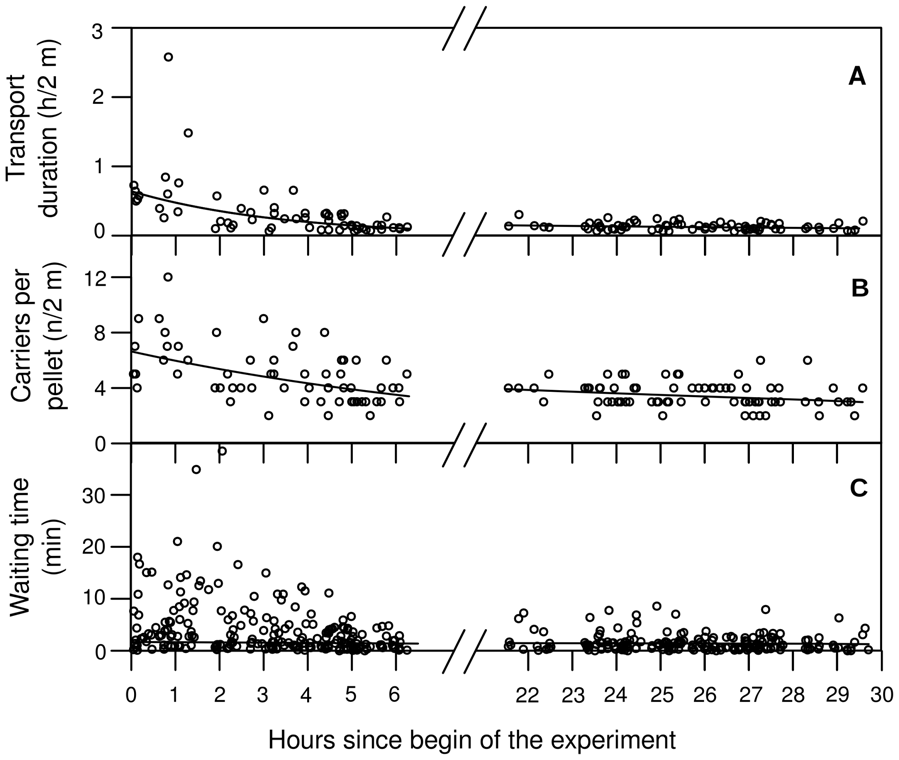
Characteristics of pellet transport as a function of time. Transport duration (d), number of carriers involved (n) and the waiting times (w) between transport events are plotted as a function of time (t). **(A)** Time needed for a pellet to be sequentially carried over a distance of 2 m from the excavation site. Solid lines show the results of regression with ln-transformed d-values on both days (first day: d = 0.6·e^−0.3·t^, *r* ^2^ = 0.45, *N* = 60, *P*<0.001; second day: d = 0.4·e^−0.04·t^, *r* ^2^ = 0.05, *N* = 73, *P* = 0.047). **(B)** The number of workers involved in the transport of single pellets. Solid lines show the results of regression with ln-transformed Y-values on both days (first day: n = 6.6·e^−0.1·t^, *r* ^2^ = 0.25, *N* = 60, *P*<0.001; second day: n = 7.9·e^−0.03·t^, *r* ^2^ = 0.06, *N* = 73, *P* = 0.039). **(C)** Time a pellet remained on the floor between two transport events. Solid lines show the results of regression with ln-transformed Y-values on both days (first day: w = 3.8·e^−0.2·t^, *r* ^2^ = 0.10, *N* = 225, *P*<0.001; second day: w = 5.5·e^−0.1·t^, *r* ^2^ = 0.02, *N* = 177, NS).

The time it took for one pellet to be transported over the whole distance of 200 cm changed during the observation period. While it took 35.2±12.0 min (median±interquartile range, *N* = 10) for pellets produced during the first hour after starting the experiment, median transport duration was 7.3±4.1 min (*N* = 73) only on the second day (Fig. 3A). The transport duration decreased as a function of time during the first day (*F*_1,58_ = 48.2, *P*<0.001) and, at a smaller rate, also at the second day (*F*_1,71_ = 4.1, *P* = 0.047).

All pellets were transported by more than one individual. Those produced during the first hour were carried by 7.0±2.5 workers (median±interquartile range, *N* = 10), while only 3.0±1.0 workers (*N* = 73) contributed to the transport of each pellet at the second day (Fig. 3B). Again, this value decreased at a higher rate during the first day (*F*_1,58_ = 19.4, *P*<0.001) than during the second (*F*_1,71_ = 4.4, *P* = 0.039).

In between two transport events, the items were simply deposited on the ground of the tube. There was no particular location for load dropping; a homogeneous layer of pellets covered the ground of the tube, this layer beginning some centimetres from the excavation site and continuing for about 30–40 cm. The ‘waiting times’ of the pellets, i.e. the time a pellet remained at one location in between two transport events, decreased on the first (*F*_1,57_ = 6.6, *P* = 0.013) and on the second day (*F*_1,71_ = 9.3, *P* = 0.003), the median being 182±263 s (*N* = 41) during the first hour, and only 64±89 s (*N* = 185) at the second day (Fig. 3C).

**Figure 4:**
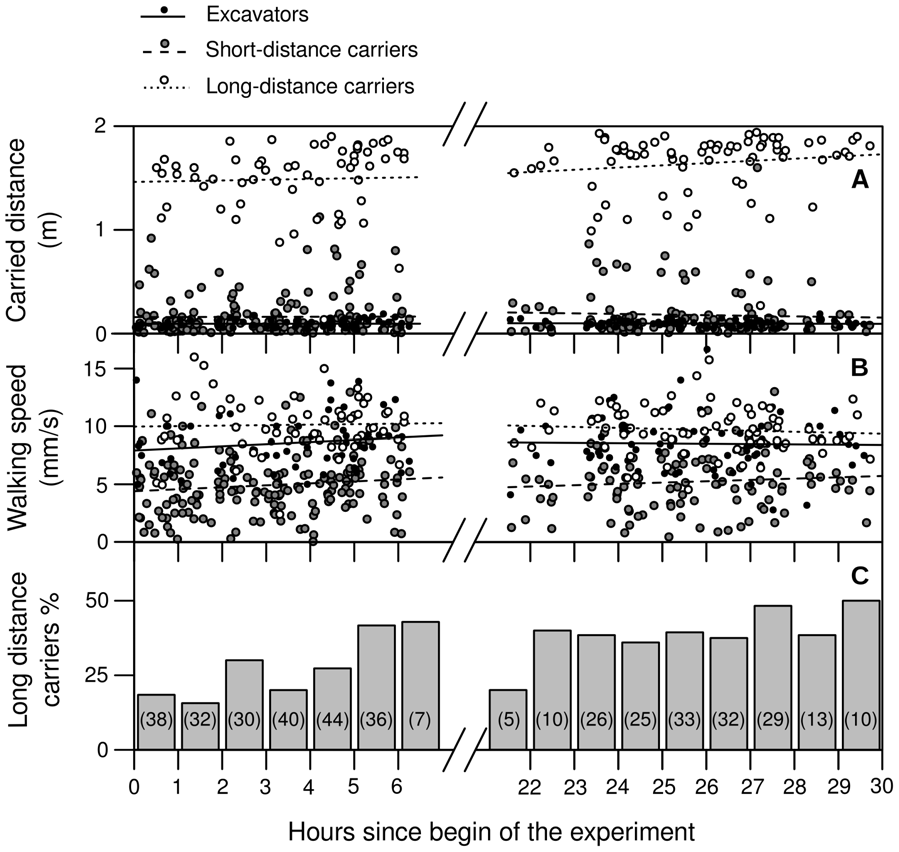
Distances walked (l) and speeds (v) measured in the different carriers involved in pellet transport as a function of time (t). Black dots and solid regression lines represent excavators, grey dots and dashed regression lines short-distance carriers, and white dots and dotted regression lines long-distance carriers. (A) Distances covered (in cm) during one transport event by excavators (first day: l = 0.53·t+7.7, NS; second day: l = −0.07·t+11.4, NS), short-distance carriers (first day: l = 0.71·t+14.0, NS; second day: l = −0.58·t+32.8, NS) and long-distance carriers (first day: l = 1.98·t+142.0, NS; second day: l = 2.13·t+108.8, NS; for 1.6% confidence intervals see Table 1). (B) Walking speed in each transport event for excavators (first day: v = 0.18·t+7.9, *r* ^2^ = 0.02, *N* = 60, NS; second day: v = −0.003·t+9.2, *r* ^2^ = 0.0005, *N* = 73, NS), short-distance carriers (first day: v = 0.17·t+4.4, *r* ^2^ = 0.01, *N* = 168, NS; second day: v = 0.11·t+2.3, *r* ^2^ = 0.008, *N* = 113, NS) and long-distance carriers (first day: v = 0.04·t+9.9, *r* ^2^ = 0.0008, *N* = 60, NS; second day: v = −0.008·t+11.9, *r* ^2^ = 0.007, *N* = 73, NS). (C) Proportion of long-distance carriers among individuals that picked up pellets. The number of observed pick-up events is shown in parentheses for each time interval.

Three different types of workers contributed to the transport of pellets. The ‘excavators’ themselves, 133 were observed during the experiment, carried their pellets for a distance of 9.5±5.5 cm (median±interquartile range, *N* = 133) until dropping them. The intermediate ‘short-distance carriers’, 281 of which were observed, transported the particles for another 9.0±14.0 cm (*N* = 281) each, until they were picked up by ‘long-distance carriers’, bringing them out into the deposition box with a significantly longer (see Table 1 for detailed statistics) median transport distance of 168.0±34.6 cm (*N* = 133) (Fig. 4A). Additionally, the three different worker types, excavators, short-distance carriers and long-distance carriers, moved at a significantly different walking speed (influence of ‘type’ on walking speed on the first day: *F*_2,286_ = 119.3, *P*<0.001; second day: *F*_2,254_ = 92.9, *P*<0.001), with the short-distance carriers moving slower than the other groups. Their average walking speed was 5.0±2.4 mm/s (*N* = 281), as compared to 8.5±2.2 mm/s (*N* = 133) for excavators, and 10.0±2.2 mm/s (*N* = 133) for long distance carriers. Walking speed did not change significantly during the observation period (influence of time on walking speed on the first day: 0.02 (mm/s)/h, *F*_1,284_ = 3.4, *P* = 0.067; second day: −0.003 (mm/s)/h, *F*_1,253_ = 0.06, *P*<0.812) (Fig. 4B).

**Table 1:** Statistics for carrying distances and temporal dynamics in the three types of workers: excavators, short-distance carriers and long-distance carriers.

Worker type	*N*	Mean distance (cm)	Regression estimate (cm/h)
**First day**			
Excavators	60	9.6^A^ (7.6 to 11.6)	0.53^NS^ (−0.33 to 1.32)
Short-distance carriers	168	16.2^A^ (7.9 to 28.1)	0.71^NS^ (−4.80 to 6.26)
Long-distance carriers	60	149.3^B^ (128.9 to 165.3)	1.98^NS^ (−7.11 to 9.47)
**Second day**			
Excavators	73	9.7^A^ (8.1 to 11.4)	−0.07^NS^ (−0.82 to 0.75)
Short-distance carriers	113	18.0^A^ (8.4 to 33.0)	−0.58^NS^ (−5.42 to 5.12)
Long-distance carriers	73	164.1^B^ (146.2 to 177.6)	2.13^NS^ (−4.83 to 8.13)

Shown are the number of observations, mean distance, and slope of the relationship between carrying distance and time, for the first and the second day separately. The 95% confidence interval for the means and the 98.3% confidence interval for the slopes, both determined by bootstrapping, are indicated in parentheses. Mean values with non-overlapping confidence intervals are considered significantly different. Values sharing the same letter are not significantly different. Slopes are considered significant if their confidence intervals do not include zeros. Significance levels are indicated in superscripts (‘NS’: not significant).

The percentage of long-distance carriers among individuals picking up pellets increased significantly during the first day (*F*_6_ = 10.2, *P* = 0.024), from 18% (*N* = 38) in the first hour to a constant proportion of 40% (*N* = 188) during the second day (Fig. 4C).

### Quantifying pellet excavation – Ethograms

The 12 individuals observed in the laboratory excavated soil pellets in relatively uniform behavioural sequences, the most marked interindividual difference being the overall period of time workers spent performing the task, ranging from 14 to 269 min with an average of 121±79 min (mean±standard deviation, *N* = 12). Within this period, the observed individuals excavated pellets at an average rate of 25.7±10.4 pellets/h (*N* = 12). During repeated sequences, the animals approached the soil and started manipulating it by what Sudd [37] has described as ‘grabbing’: the mandibles were inserted into the clay, a behaviour that, in the observed case of excavating in wet clay, much resembles cutting behaviour in other materials. The workers virtually bite into the tough material. After that, the soil was ‘raked’ together by using both forelegs and the closed mandibles that were used much like an ice-cream scoop to aggregate soil beneath the body in an anterioposterior pulling movement. After one or more sequences of grabbing and raking, the accumulated body of soil was picked up with the mandibles, carried away and dropped at another location. The average time spent to form one pellet was 41.0±30.0 s (*N* = 536). After dropping the pellet the worker had produced itself, it occasionally picked up one or two other pellets and relocated them before starting to excavate the next pellet. Thus, between two consecutive excavation sequences workers relocated between one and three pellets. Therefore, besides producing the pellets, excavators also relocated them at a mean rate of 25.8±6.2 pellets/h (Table 2).

**Table 2:** Long-term observations of individual excavators.

Ant	Time (min)	Dug	Carried	Work rates (pellets/h)		Digging seq	Dropping location			DI
				Digging	Transport	(s/pellet)	Arena	Tube	Box	
1	14	5	4	21.9	17.5	28.0±20.4	0	3	1	—
2	180	63	60	21.0	20.0	61.0±40.1	1	1	58	—
3	269	71	88	15.9	19.6	35.7±23.0	8	1	79	—
4	78	29	32	22.4	24.7	51.8±40.0	1	0	31	6.0
5	180	63	57	21.1	19.0	37.4±32.0	3	13	41	9.0
6	189	85	98	27.0	31.1	41.7±32.4	3	0	95	6.2
7	44	21	26	28.5	35.3	29.4±15.0	0	3	23	7.2
8	64	29	27	27.2	25.3	46.0±30.1	1	2	24	6.6
9	156	65	67	25.1	25.8	40.6±23.2	1	1	65	8.3
10	150	41	72	16.4	28.8	33.1±18.3	0	20	51	8.3
11	18	17	11	56.3	36.4	33.2±25.4	11	0	0	—
12	108	47	47	26.0	26.0	32.5±17.4	0	1	46	6.7

Shown are, for each of the 12 observed workers, the duration of its continuous excavation activity, the number of pellets excavated and the number of pellets carried within that time, the resulting digging and transport rates, the average time spent for excavating a single pellet (mean±SD), the locations where the pellets were deposited and, for those pellets deposited in the box, the Index of Dispersion (DI) for their distribution on the 16 square areas. A DI close to 0 is an indicator of a regular distribution. Values around 0.5 characterise random distributions, while values close to 1 and higher indicate an aggregated distribution. For four individuals (1, 2, 3 and 11) not every pellet dropping could be observed, and therefore no dispersion indices were assigned to them.

**Figure 5:**
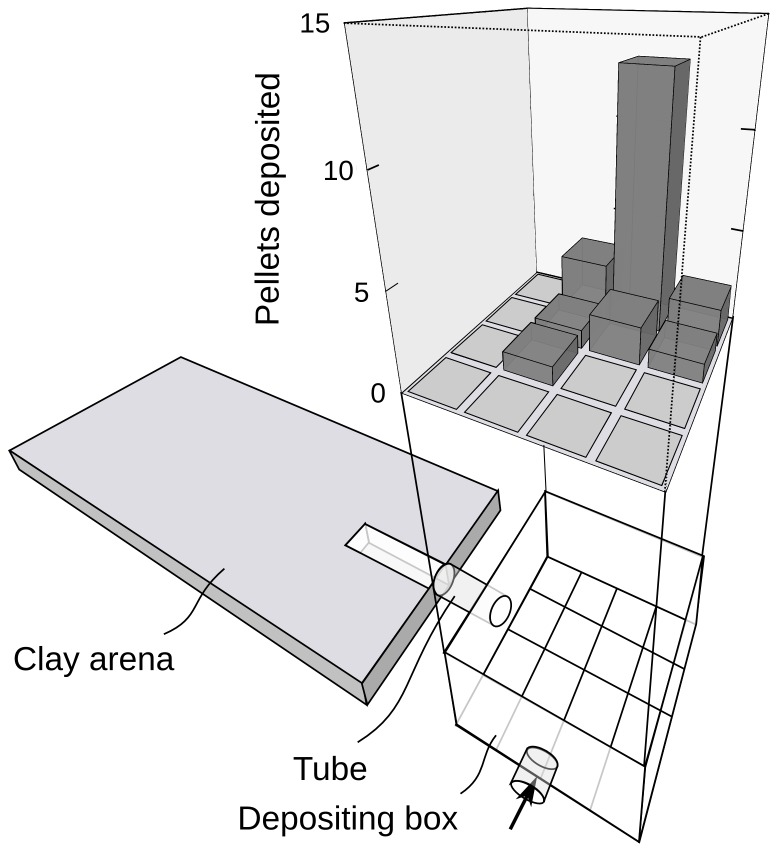
Example of the distribution of pellets deposited by a single worker in the depositing box. Shown are the clay arena, where individually marked workers excavated soil pellets, and the depositing box, where most pellets were dropped by the observed individuals. The thick arrow indicates the location where the animals entered the set-up. To reconstruct the individual pattern of pellet deposition for each observed worker, the ground of the depositing box was divided into 16 equal squares. The number of pellets dropped in each square by one individual was counted. Each bar in the graph represents one of the possible 16 areas distinguished inside the box, with the height of the bar indicating the number of pellets dropped inside this field by the observed individual. The worker observed for this example (worker nr. 7) placed 23 pellets in the box within a period of 44 min. The resulting index of dispersion was 7.2. The three-dimensional barplot is based on a script by Michal J. Figurski (http://addictedtor.free.fr/graphiques/graphcode.php?graph=161).

The majority of pellets were relocated directly into the depositing box. Within the depositing box, the pellets appeared to be evenly distributed on the ground without apparent aggregations. However, at the individual level workers preferred to drop all their pellets within one particular area. As the high Indices of Dispersion, averaging 7.3±1.1 (*N* = 8), indicate, all the pellets carried by a single individual were not dropped in the box randomly, but placed in an aggregation (Fig. 5), each worker aggregating its own loads at a different place.

### Dropped pellets as cues for digging decisions – Choice experiments

**Figure 6:**
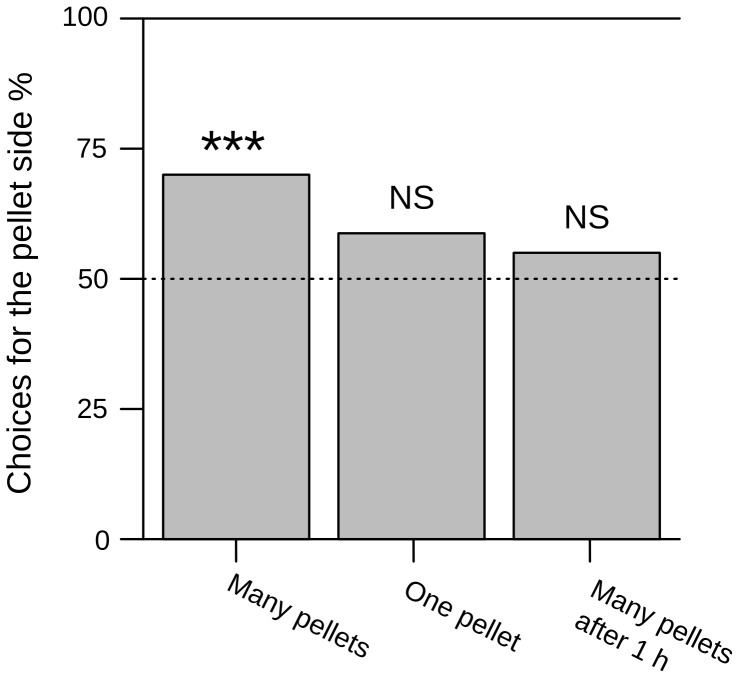
Choices between two alternative locations to excavate. To different locations were offered for excavation. At one side, pellets were deposited on the ground (see Fig. 1). The sample size was 80 for all conditions. Significance levels for binomial tests are indicated above the bars (‘NS’ not significant, ‘*’ *P*<0.05, ‘**’ *P*<0.01, ‘***’ *P*<0.001).

Using a layer of fresh pellets as a stimulus in the choice situation, excavation was first initiated at the side where the pellets were presented in 70% (56 of 80) of the experiments. The ants significantly (*N* = 80, *P*<0.001) preferred to dig at that side. If pellets were offered as stimulus after a waiting period of one hour, there was no significant effect: the side with the pellet layer was chosen in 59% (47 of 80) of the trials (N = 80, P = 0.146). With only one fresh pellet as a stimulus, the corresponding side was chosen in 55% (44 of 80) of the trials and again, the effect was not significant (N = 80, P = 0.434) (Fig 6).

## Discussion

Our results indicate that underground soil transport strongly resembles the long-distance sequential transport of leaf fragments by foraging leaf-cutting ants, and that task partitioning during transport results in accumulations of fresh pellets that provide a cue influencing other workers in their decision where to excavate. Single pellets were not carried to the surface by the excavator directly, but transported by several individuals and deposited on the way in between transport events. Excavators themselves carried their pellets for about 9.5 cm only and they were selective in choosing a location where to drop them, usually aggregating their pellets individually at one spot. Several short-distance carriers picked the items up and carried them at low speeds over short ranges before dropping them on the ground. Finally, the long-distance carriers picked up the loads again, and covered the rest of the way until the final deposition outside the nest. The number of short-distance carriers contributing to the transport of each pellet was higher during the establishment of excavation activity at a new location, and dropped over time, indicating that more workers carried the pellets directly to the outside as the digging process progressed.

During soil transport, load size was shown to depend on carrier size as also reported for leaf transport [38]. This effect was only observed in carriers, but not in excavators, which walk only very short distances before returning to the excavation site. Size matching in carriers suggests that they are either highly selective when picking up pellets for transport, or actively manipulate the sticky material to create a load roughly adjusted to their body size. This may suggest that task partitioning among carriers facilitates size-matching, and is therefore aimed at maximizing material transport rates, as discussed for leaf foraging [24], [25], [39], even though we did not find direct support for this hypothesis because excavators and long distance carriers walked at the same speed. It is important to mention that the loading ratios observed in the field were larger than those from the laboratory. While workers in the laboratory carried loads roughly equivalent to their body mass, field workers carried loads that were on average three times heavier than their bodies, a phenomenon that may be related to the physical properties and/or humidity of the heterogeneous natural soils that influence the aggregation of the material to be carried as a pellet.

Sequential task partitioning in foraging *A. vollenweideri* workers follows a similar pattern [28]. As described for the excavators, cutters contribute little to the transport of the leaf fragment they harvested. Short-distance carriers cover small portions of the overall distance when the leaf fragment is close to its origin, until a long-distance carrier picks the item up to carry it for the rest of the way to the nest. Again, there is no particular location on the way for dropping. Leaf fragments are transported significantly slower in transport chains than when carried directly by one worker only [28], [29].

Two hypotheses have been suggested to account for the advantages of sequential transport in foraging leaf-cutting ants [8], [28], [29], [39]. First, sequential load transport may have been favoured during evolution because of a faster load delivery rate. These arguments are the core of the so-called ‘economic-transport hypothesis’, with ‘economic’ in this context referring to the maximization of the transportation speed of a leaf fragment, which at the colony level may result in an increased overall rate of resource delivery [28]. A faster material transport rate may be achieved via improved size-matching, as discussed above, or because each contributing worker restricts its task to a certain part of the way, which may improve its orientation and, indirectly, its walking speed. It remains an open question whether a worker harvesting a leaf fragment may succeed in carrying it back to the nest for about 100 m, and then in finding its way back to the cutting site. If not, sequential transport would be absolutely necessary to exploit distant leaf patches as rapidly as possible. Second, leaf fragments may inform other foragers either about type and quality of a newly exploited food patch, or simply about the fact that such a food patch can be found in that direction. In this case, the behavioural response of transferring fragments would have been selected for because of its positive effect on the information flow. This ‘information-transfer hypothesis’ states that workers may trade off material transport rate for enhanced information transfer during social foraging, and field experiments on foraging *Atta vollenweideri* leaf-cutting ants provided support for these arguments [29].

With regard to the described similarities between leaf transport and soil transport, both hypotheses might be valid for soil transport as well. We observed task partitioning between short-distance and long-distance carriers. Both groups differed not only in the distances they covered, but also in their carrying speed, which was significantly slower in short-distance carriers. We can only speculate about the function of short-distance carriers during soil transport. Possibly, the parts of the tunnel where pellets are deposited constitute a challenge to the ants' orientation abilities. The constant loading and unloading activity may result in a permanent change of chemical and physical cues, requiring specialized individuals, each familiar with a short section of the changing local environment, to carry the loads. Slow walking speeds of short-distance carriers may result from slow orientation movements and/or walking difficulties on a pellet-covered surface. Another possibility to account for the observed slow walking speed is that short-distance carriers move at a slower pace to contact workers and so to enhance the information flow about the current digging activity. Encounter rates with workers carrying a pellet might inform nest mates about current digging activity, in addition to the effect of deposited pellets on workers' digging choices. Interaction patterns are known to influence task allocation in insect societies, by informing individuals about the activities of nest mates [40]. Hypothetically, encounter rates with soil carriers can inform workers about both the fact that nest mates are excavating and the direction leading to the place where nest enlargement is happening. The fact that the proportion of short-distance carriers contributing to the transport of each pellet decreases within the first few hours, as shown in our experiments, appears to support the hypothesis that they may contribute to the recruitment of nestmates to the excavation site, for recruitment would be more important when excavation activity begins at a new location than later on. Finally, our observations indicate that in between two excavation events, some excavators occasionally pick up other pellets and relocate them. We need therefore to assume that a significant proportion of the short-distance carriers observed in our ‘pelletograms’ were in fact excavators temporarily working as carriers. Since excavators are known to actively recruit nest mates to the digging site by producing stridulation signals while forming their pellets [41], their behaviour as short-distance carriers, and the spatial arrangement of the carried pellets, could also be considered as part of the recruitment system.

In our experiments, excavators formed pellets at a relatively constant rate for an average period of 2 h. They deposited their pellets close to the digging site and were highly selective about where they were dropped. With several excavators working in parallel, individual deposition patterns resulted in a homogeneous layer of pellets covering the ground nearby the excavation site. The observations that individuals aggregate their own pellets, but no obvious aggregation results at the group level, may appear contradictory if we expect pellets to act as stigmergic cues. But they can be interpreted in the terms of maximizing individual working rates: the individual selectivity of dropping points by the excavators may improve their individual performance by allowing them to quickly return and to find their excavation site.

We demonstrated in our choice experiments that a number of fresh pellets deposited close to a potential digging site increases the probability of other workers to prefer it over an alternative site when deciding where to dig. It is therefore likely that primary underground deposits of soil pellets act as stigmergic cues. It has been recently demonstrated in the same species that stridulation signals produced by digging individuals attract nestmates to excavate at the same location [41]. The advantage of one individual preferring to excavate close to other individuals is the possibility of a spatial organisation and coordination of collective digging activity: many individuals choose the same location to excavate, which results in a faster emergence of a larger structure. Pellets deposited close to excavation sites, which were shown in the present study to be attractive to other workers and to act as stigmergic cues, may have a comparable effect. Being spread out along the tunnel for up to 40 cm, they may even complement stridulation signals, which are effective only at distances shorter than 6 cm [41].

Our results indicate that the effect of deposited pellets as a cue depends on two variables: quantity and time since its excavation. A single pellet in the tunnel had no measurable effect on the worker's decision as to where to excavate. If we hypothesize that sequential transport of soil pellets has been favoured during evolution to provide cues aimed at aggregating excavators at one spot, a large number of pellets may more likely originate from a large number of excavators, and should therefore be more attractive as a stigmergic cue. The second variable important for the effectiveness of deposited pellets as a cue was the time since its excavation. After one hour, the pellets, even in large numbers, had lost their effect in the choice experiment. It remains an open question if this was due to chemicals on the pellet surface that evaporated in this period. Only two cases have been reported in which pheromones induced excavation behaviour in ants [42], [43]. In both cases, the substances were described as alarm pheromones and their use during regular nest excavation was not further investigated. However, both substances were extracted from the mandibular gland: hypothetically, they could be applied onto a pellet while digging without any marking behaviour obvious to the observer. Another possibility would be that saliva or traces of cuticular hydrocarbons from the excavator's mandibles remain on the pellet only for some time after excavation.

Whatever the mechanisms leading to the lack of attractivity over time, responding only to fresh pellets as cues allows workers to join the current excavation activity. It is due to task partitioning in the process of soil transport that excavated pellets are left on the way to the excavation site, thereby providing cues influencing the excavation behaviour of nearby workers. As a consequence, one important advantage of sequential transport, besides its potential benefits for the orientation in the complex environment of a giant underground nest, can be seen in providing these cues. Hereby, task partitioning during soil transport likely contributes to the spatial organisation of collective excavation behaviour that leads to the complex architecture of leaf-cutting ant nests.

## References

[1] Hansell M (2005) Animal Architecture. New York: Oxford University Press.

[2] Emerson AE (1938) Termite nests - a study of the phylogeny of behavior. Ecological Monographs 8: 247–284.

[3] Weber NA (1972) Gardening Ants: The Attines. Philadelphia: The American Philosophical Society.

[4] Jarvis JUM, Sale JB (1971) Burrowing and burrow patterns of East African mole-rats *Tachyoryctes*, *Heliophobius* and *Heterocephalus* . Journal of Zoology 163: 451–479.

[5] Jeanne RL (1986) The organization of work in *Polybia occidentalis*: costs and benefits of specialization in a social wasp. Behavioral Ecology and Sociobiology: 333–341.

[6] Jeanne RL (1986) The evolution of the organization of work in social insects. Monitore Zoologico Italiano 20: 119–133.

[7] Anderson C, Ratnieks FLW (2000) Task partitioning in insect societes: novel situations. Insectes Sociaux 47: 198–199.

[8] Anderson C, Boomsma JJ, Bartholdi JJ (2002) Task partitioning in insect societies: bucket brigades. Insectes Sociaux 49: 171–180.

[9] Cosarinsky MI, Roces F (2012) The construction of turrets for nest ventilation in the grass-cutting ant *Atta vollenweideri*: import and assembly of building materials. Journal of Insect Behavior 25: 222–241.

[10] Hingston RWG (1929) Instinct and Intelligence. New York: The Macmillan Company..296 p.

[11] Kleineidam CJ, Roces F (2000) Carbon dioxide concentrations and nest ventilation in nests of the leaf-cutting ant *Atta vollenweideri* . Insectes Sociaux 47: 241–248.

[12] Kleineidam CJ, Ernst R, Roces F (2001) Wind-induced ventilation of the giant nests of the leaf-cutting ant *Atta vollenweideri* . Naturwissenschaften 88: 301–305. 11544898 10.1007/s001140100235

[13] Cosarinsky MI, Roces F (2007) Neighbor leaf-cutting ants and mound-building termites: Comparative nest micromorphology. Geoderma 141: 224–234.

[14] Jonkman JCM (1980) The external and internal structure and growth of the nests of the leaf-cutting ant *Atta vollenweideri* Forel, 1893 (Hym. : Formicidae) Part I. Zeitschrift für angewandte Entomologie 89: 158–173.

[15] Jonkman JCM (1980) The external and internal structure and growth of the nests of the leaf-cutting ant *Atta vollenweideri* Forel, 1893 (Hym.: Formicidae) Part II. Zeitschrift für angewandte Entomologie 89: 217–246.

[16] Bucher EH, Zuccardi RB (1967) Significación de los hormigueros de *Atta vollenweideri* Forel como alternadores del suelo de la provincia de Tucumán. Acta Zoologica Lilloana 23: 83–95.

[17] Jonkman JCM (1976) Biology and ecology of the leaf-cutting ant *Atta vollenweideri* Forel, 1893. Zeitschrift für angewandte Entomologie 81: 140–148.

[18] Jonkman JCM (1978) Nests of the leaf-cutting ant *Atta vollenweideri* as accelerators of succession in pastures. Zeitschrift für angewandte Entomologie 86: 25–34.

[19] Sosa B, Brazeiro A (2010) Positive ecosystem engineering effects of the ant *Atta vollenweideri* on the shrub *Grabowskia duplicata* . Journal of Vegetation Science 21: 597–605.

[20] Délye G (1957) Observations sur la fourmi Saharienne *Cataglyphis bombycina* Rog. Insectes Sociaux 4: 77–82.

[21] Délye G (1971) Observations sur le nid et the comportement constructeur de *Messor arenarius* (Hyménotères Formicidae). Insectes Sociaux 18: 15–20.

[22] Spangler HG, Rettenmeyer CW (1966) The function of the ammochaetae or psammophores of harvester ants, *Pogonomyrmex* spp. Journal of the Kansas Entomological Society 39: 739–745.

[23] Sleeman J, Brewer R (1972) Microstructures of some Australian termite nests. Pedobiologia 12: 347–373.

[24] Lighton JRB, Bartholomew GA, Feener DHJ (1987) Energetics of locomotion and load-carriage and a model of the energy cost of foraging in the leaf-cutting ant *Atta colombica* Guer. Physiological Zoology 60: 524–537.

[25] Röschard J, Roces F (2002) The effect of load length, width and mass on transport rate in the grass-cutting ant *Atta vollenweideri* . Oecologia 131: 319–324. 28547700 10.1007/s00442-002-0882-z

[26] Fowler HG, Robinson S (1979) Foraging by *Atta sexdens* (Formicidae: Attini): seasonal patterns, caste and efficiency. Ecological Entomology 4: 239–247.

[27] Hubbell SP, Johnson LK, Stanislav E, Wilson B, Fowler HG (1980) Foraging by bucket-brigade in leaf-cutter ants. Biotropica 12: 210–213.

[28] Röschard J, Roces F (2003) Cutters, carriers and transport chains: distance-dependent foraging strategies in the grass-cutting ant *Atta vollenweideri* . Insectes Sociaux 50: 237–244.

[29] Röschard J, Roces F (2011) Sequential load transport in grass-cutting ants (*Atta vollenweideri*): maximization of plant delivery rate or improved information transfer? Psyche: A Journal of Entomology 2011: 1–10. Available: http://www.hindawi.com/journals/psyche/2011/643127/. Accessed 27 March 2012.

[30] Autori M (1942) Contribuição para o conhecimento da saúva (*Atta* spp. Hymenoptera - Formicidae). III. Excavação de um saúveiro (Atta sexdens rubropilosa Forel, 1908). Arquivos do Instituto Biológico 13: 137–148.

[31] Fröhle K, Roces F (2009) Underground agriculture: the control of nest size in fungus-growing ants. In: Theraulaz G, Solé R, Kuntz P, editors. From Insect Nests to Human Architecture - Workshop on Engineering Principles of Innovation in Swarm-made Architectures. European Centre for Living Technology, Venice, Italy, pp. 95–104.

[32] Fröhle K (2009) Mechanismen zur Regulierung der Nestgröße während des Koloniewachstums bei Blattschneiderameisen. PhD-Thesis, Julius-Maximilians-Universität Würzburg. Available: http://www.opus-bayern.de/uni-wuerzburg/volltexte/2010/4631/pdf/Froehlediss.pdf.Accessed 29 July 2010.

[33] Grassé P-P (1959) La reconstruction du nid et les coordination inter individuelles chez *Bellicositermes natalensis* et *Cubitermes* sp. La théorie de la stigmergie: Essai d’interprétation du comportementdes termites constructeurs. Insectes Sociaux 6: 41–81.

[34] Theraulaz G, Bonabeau E (1999) A brief history of stigmergy. Artificial Life 5: 97–116. 10633572 10.1162/106454699568700

[35] Freedman D (1981) Bootstrapping regression models. The Annals of Statistics 9: 1218–1228.

[36] Lee S, Bardunias P, Su N-Y, Yang R (2008) Behavioral response of termites to tunnel surface irregularity. Behavioural Processes 78: 397–400. 18359581 10.1016/j.beproc.2008.02.009

[37] Sudd JH (1969) Excavation of nests by ants. Zeitschrift für Tierpsychologie 26: 257–276.

[38] Röschard J, Roces F (2003) Fragment-size determination and size-matching in the grass-cutting ant *Atta vollenweideri* depend on the distance from the nest. Journal of Tropical Ecology 19: 647–653.

[39] Roces F, Bollazzi LM (2009) Information transfer and the organization of foraging in grass- and leaf-cutting ants. In: Jarau S, Hrncir M, editors. Food Exploitation by Social Insects: Ecological, Behavioral and Theoretical Approaches. Boca Raton, USA: CRC Press: Contemporary Topics in Entomology Series. pp. 261–275.

[40] Gordon D (1996) The organization of work in social insect colonies. Nature 380: 121–124.

[41] Pielström S, Roces F (2012) Vibrational communication in the spatial organization of collective digging in the leaf-cutting ant *Atta vollenweideri* . Animal Behaviour 84: 743–752.

[42] Wilson EO (1958) A chemical releaser of alarm and digging behavior in the ant *Pogonomyrmex badius* (Latreille). Psyche 65: 41–51.

[43] Blum MS, Warter SL (1966) Chemical releasers of social behavior. VII. The isolation of 2-Heptanone from *Conomyrma pyramica* (Hymenoptera: Formicidae: Dolichoderinae) and its modus operandi as a releaser of alarm and digging behavior. Annals of the Entomological Society of America 59: 774–779.

